# Parathyroid Hormone-Related Protein (PTHrP): A Key Regulator of Life/Death Decisions by Tumor Cells with Potential Clinical Applications

**DOI:** 10.3390/cancers3010396

**Published:** 2011-01-20

**Authors:** Claudio Luparello

**Affiliations:** Dipartimento di Biologia Cellulare e dello Sviluppo, Università di Palermo, Viale delle Scienze, 90128 Palermo, Italy; E-Mail: claudio.luparello@unipa.it; Tel.: +39 091 23897405; Fax: +39 091 6577430

**Keywords:** PTHrP, cell proliferation, apoptosis, tumor cells

## Abstract

Parathyroid hormone-related protein (PTHrP), classically regarded as the mediator of the humoral hypercalcemia of malignancy syndrome, is a polyhormone that undergoes proteolytic processing into smaller bioactive forms. These bioactive forms comprise an N-terminal-as well as midregion-and C-terminal peptides, which have been shown to regulate various biological events, such as survival, proliferation and differentiation, in diverse cell model systems, both normal and pathological. A number of experimental data have demonstrated that PTHrP is also able to modulate tumor-relevant phenotypic expressions, thereby playing a role in early and advanced tumorigenesis, and in the response to treatment. In particular, interest has mainly been focused on the effects of PTHrP on cell proliferation/apoptosis, migration and invasion, which are the main roles involved in cancer development *in vivo*. The objective of this review is to discuss collectively the literature data on the molecular and biochemical basis of the mechanisms underlying the different, and sometimes opposite, effects exerted by PTHrP on various neoplastic cytotypes, with some final comments on both present and potential utilization of PTHrP as a target for anti-cancer therapy.

## 1. Introduction: PTHrP is a Multidomain and Multifunctional Protein

Parathyroid hormone-related protein (PTHrP), classically regarded as the mediator of the humoral hypercalcemia of malignancy syndrome, is the product of a gene spanning more than 15 kb of genomic DNA and exhibiting a complex organization in humans, where it generates multiple mRNA variants through alternative splicing events and utilization of different promoters. The initial translation products are three isoforms of 139, 141, and 173 amino acids with distinct C-terminals, displaying sequence homology with PTH at the extreme N-terminus, which allows the binding to the same G protein-linked receptor PTH1R. Indeed, PTHrP is a prohormone, the post-translational processing of which generates a family of mature secretory forms of the peptide. Apart from PTHrP (1–36), which contains homology with PTH and is able to activate the PTH/PTHrP receptor, a midregion fragment, *i.e.*, PTHrP (38–94), and a C-terminal fragment of PTHrP surmised to be comprised of PTHrP (107–139), have also been identified as mature secretory forms of the protein. It is now known that the latter acts on skin, heart and bone cells. PTHrP (38–94), whose cell receptor is still unknown, is able to activate intracellular Ca^++^ pathways at low concentrations, being critical for driving Ca^++^ transport from the maternal to the fetal circulation through the placenta, which is the sole normal tissue in which this PTHrP domain has been shown to exert an effect [1]. Noteworthy, midregion PTHrP has also been shown to exert effects in malignant tissues [2], which will be discussed in greater detail later. It is widely-acknowledged that midregion PTHrP possesses a lysine/arginine-rich bipartite sequence, encompassing amino acids 87–107, which is homologous to the nuclear/nucleolar targeting signal (NTS) present in SV40 large tumor antigen, able to direct importin β/Ran GTPase-mediated import thereby allowing the peptide to play the so-called “intracrine” role that supplements the “autocrine/paracrine” one. In the case of intracrine pathway, the endogenous PTHrP is synthesized starting from alternative translation start sites bypassing the PTHrP N-terminal signal sequence. The translated protein cannot enter the endoplasmic reticulum, but translocates to the nucleoplasm due to its NTS [3]. Several published papers have reported the activity of PTHrP or its discrete domains as inducers of cell proliferation or, conversely, cell death and apoptosis in non pathological model systems [4-7].

Interestingly, one of the original tumors from which PTHrP was purified and sequenced, because of the elevated concentration of its circulating form, was breast cancer [8,9] and it is now well-known that PTHrP exerts multiple effects on different neoplastic cytotypes via cytosolic and nuclear targets, thereby participating to initiate and promote tumor growth and dissemination [10]. It is known that PTHrP participates in various complex signaling pathways via its membrane and nuclear effects: evidence exists that the protein and/or its discrete fragments can activate protein kinase C and A routes as well as protein G βγ dimer-mediated mechanisms and that p21, AKT and NFκB are among the final targets of such signals [11]. The present mini-review is focused on the role of PTHrP as a life/death regulator in animal and human tumor cell models, with particular interest on the biochemical and molecular mechanisms involved, providing also some examples of present and potential utilization of PTHrP as a target for anti-cancer therapy.

## 2. PTHrP as an Anti-Proliferative and Death-Promoting Factor

### 2.1. PTHrP and Osteosarcoma Cells

In 1997, Valin and co-workers [12] demonstrated the growth inhibitory effect of the PTHrP (107–111) and (107–139) domains on the rat osteosarcoma cell line UMR 106 through a protein kinase C- (PKC) dependent mechanism. This effect, ascribed to the sole C-terminal fragments, was apparently mediated by a then-unknown receptor, different from the “classical” PTH/PTHrP receptor, binding the *N*-terminal sequence shared by the two hormones. It is now strongly suggested that C-terminal PTHrP intracellular signaling occurs via the putative TRSAW receptor, recognizing to the Thr^107^ Arg^108^ Ser^109^Ala^110^ Tyr^111^ portion of the peptide, which is able to activate membrane–bound PKC activity but not the adenylate cyclase [13]; interestingly, a similar effect was also recorded in rodent and human skin keratynocytes. In addition, PTHrP (107–111) was also found able to induce the intracellular Ca^++^ signal via transient opening of L-type Ca^++^ channels, thereby affecting the expression of genes whose promoters contain Ca^++^-responsive elements and/or other intracellular pathways such as those dependent upon Ca^++^-calmodulin [1,10,14].

### 2.2. PTHrP and MDA-MB231 Breast Cancer Cells

In 1995, Luparello and collaborators [15] tested the effect of different PTHrP domains on the proliferative behavior of 8701-BC, a cell line derived from a primary ductal infiltrating carcinoma (d.i.c.) of the human breast and thereby being more representative of the heterogeneity present in the tumor cell population *in vivo* [16]. The results obtained indicated that PTHrP (67–86) and, more prominently (107–138) and (1–34), exerted anti-proliferative but pro-invasive effects to different degrees, which were abolished by incubation with anti-PTHrP antibody. In addition, experiments with clonal cell lines isolated from the parental 8701-BC line and endowed with different proliferative and invasive properties *in vitro* demonstrated the heterogeneity of the growth and invasive response of the different subpopulations to administration of PTHrP fragments, thereby suggesting the existence of complex PTHrP-breast cancer cell interplays in the affected tissue [17]. Further data obtained after the exposure of 8701-BC cells to PTHrP (67–86) indicated the effect on the modulation of gene expression, in particular identifying *heat shock factor binding protein-1* and *90 kDa-heat shock protein* as the up-regulated genes. In turn, such over-expression was found to be involved in the modulation of the expression of *urokinase-plasminogen activator* and *matrix metalloprotease-1* and, consequently, in the acquisition of an invasive behavior *in vitro* by this cell line [18].

Another set of data was obtained using the estrogen receptor (ER)-negative and highly malignant MDA-MB231 breast cancer cell line as a model system. In Luparello and coworkers' paper in 2001 [19], cell viability, proliferation, invasiveness and growth in nude mice was examined following administration of the midregion (38–94) fragment of PTHrP, which was proven to reduce markedly breast cancer growth and invasion *in vitro* and *in vivo*, thereby representing an attractive target for molecular modeling of smaller, orally active anti-neoplastic agents. Subsequent studies revealed that this midregion peptide is imported in the cell nucleoplasm and is endowed with DNA-binding properties *in vitro*, thus potentially acting as a transcription factor-like molecule. An effect at the gene expression level was reported, especially addressed to the regulation of some apoptosis-related genes (*Bad*, *Bcl-xS*, *Receptor-interacting protein 1*, *caspase-2*, *-5*, *-6*, *-7* and *-8*) that may be responsible, at least in part, for the lethal effect imparted by PTHrP (38–94) on MDA-MB231 cells [20-22].

Interestingly, some opposite results, *i.e.*, growth-promoting, were found using the ER-positive MCF-7 cell line. In particular, in 2009, Alokail [23] investigated the effect of different N-terminal PTHrP domains on epidermal growth factor receptor (EGFR)-transfected cells and demonstrated that the receptor induced ERK activation via heregulin β1 and PTHrP and both ERK and PKC induced the mitogenic effects via MAPKs, thereby potentially explaining the PTHrP-dependent proliferative effects.

### 2.3 PTHrP and Lung Cancer Cells

Although listed among the anti-proliferative and death-promoting examples, conclusions on the effect of PTHrP on lung cancer cells cannot be drawn as in the literature this topic is still under discussion. In 1990, Burton and collaborators [24] reported the growth stimulatory effect of PTHrP (1–34) on BEN squamous cell lung carcinoma cells *in vitro*, whereas in 2001, Hastings and co-workers [25] demonstrated that the peptide inhibits the growth of the same cells and that exposure to anti-PTHrP antibody stimulated the growth of BEN cell carcinomas in athymic mice, thereby highlighting the paracrine growth inhibitory effects of N-terminal PTHrP fragment on lung tumor. This discrepancy is probably a consequence of clonal heterogeneity of the cell line. Moreover, anti-apoptotic effects of PTHrP (1–34) and (140–173) on BEN cells have been reported [26] and could contribute to lung cancer progression. Interestingly, Hastings and coworkers [27] have also documented that cyclic thiourea compounds inhibit *PTHrP* expression mediated by the P3 promoter in BEN cells and that they may inhibit growth of lung cancer cells through the same mechanism. A patient population study [28] reported the longer survival of women affected with PTHrP-secreting lung carcinomas; in addition, more recently Monego and collaborators [29] showed that the expression of both PTHrP (1–34) and PTH1R are independent prognostic markers of a worse clinical outcome in lung adenocarcinoma patients.

When PTHrP production-lacking lung adenocarcinoma cells, transfected with with a pciNeo-PTHrP 1–87 expression plasmid, were examined for their growth and intracellular signalization aspects, the reduced mitogenesis observed was found linked to a block in G_1_ and, from a molecular point of view, to the decreased expression of cyclin D2 and cyclin A2, increased expression of p27, decreased association of cyclin A2 and CDK2, and increased activation of ERK [30]. The authors, therefore, stressed the necessity of further investigation to explore this promising association between N-terminal PTHrP, slowing of tumor progression and increased patient survival. Noteworthy, the cited works did not consider the acknowledged intracrine effect mediated by PTHrP NTS, whose impact on lung cancer cell viability and proliferation awaits further investigations.

## 3. PTHrP as a Stimulator of Cell Survival and Proliferation of Tumor Cells: A Promising Target for Therapeutic Intervention

A number of experimental data obtained on different neoplastic model systems have brought evidence that PTHrP is a pro-survival, anti-apoptotic and proliferation-promoting factor, thereby highlighting the potential therapeutic benefit of the modulation of PTHrP production.

Already in 1995, in fact, Rabbani and coworkers [31] had observed that the application of PTHrP antisense strategy to an animal model of Leydig cell tumor produced a significant decrease of doubling time *in vitro* and the lowering of tumor volume when antisense-transfected cells were inoculated into recipient rats. Few years later, PTHrP was shown to exert a positive influence also on the size of primary prostate carcinoma in rats and its protection against apoptotic stimuli on neoplastic cells was first suggested [32]. Dealing with MCF-7 ER-positive breast cancer cells, in 2000, Falzon and Du [33] proposed that “intracrine” and “autocrine/paracrine” pathways could transduce opposite instructions to cells, the former being proliferation-restraining whereas the latter inducing cell growth, thus adding a further level of complexity to the biological responses of cells to PTHrP. Additional experiments on the effect of intracrine signalization demonstrated that over-expression of wild-type *vs.* NTS-mutated PTHrP determined the increase of the percentage of cells in G_2_/M phases of the cell cycle, and Bcl-2/Bax and Bcl-xL/Bax ratios, indicative of protection from apoptosis [34].

More recently, investigation on the mechanism of action of PTHrP has been focused mainly on prostate, colon and renal cancer cells, although scattered reports of interest on chondrosarcoma, medulloblastoma, anaplastic thyroid and adrenocortical tumor cells have also appeared. Literature data can be summarized as follows.

### 3.1. PTHrP and Chondrosarcoma Cells

In 2003, Miyaji and collaborators [35] were the first to investigate the effects of treatment with anti-PTHrP monoclonal antibodies on cell viability and differentiation of chondrosarcoma HTB-94 cells, finding that the treatment triggered apoptosis via, at least in part, imbalance of Bcl-2/Bax ratio, activation of caspase-3 and PARP cleavage, also accelerating cell differentiation to the hypertrophic chondrocyte stage. Since apoptosis and differentiation are both instrumental in reducing chondrosarcoma malignancy, the authors concluded that antibody treatment could be an alternative and less aggressive option, still to be tested *in vivo*, to surgical resection.

### 3.2. PTHrP and Anaplastic Thyroid Cancer Cells

It is known that human anaplastic thyroid cancer cells produce PTHrP which is a likely contributor to the dedifferentiated and aggressive phenotype shown by such tumor cells. In 2005, Dackiw and coworkers [36] reported that the farnesyl transferase inhibitor manumycin A, which is a microbial product, was active in both decreasing nuclear import of PTHrP and increasing proteasome-mediated PTHrP degradation by this tumor cytotype, and displayed an inhibitory and pro-apoptotic effect *in vitro* and *in vivo*. Since the reduction of intracellular PTHrP may be involved in the anti-neoplastic effect of manumycin, the authors suggested the importance of gaining more insight in the mechanism of modulation of PTHrP levels and on its potential therapeutic effect on this lethal malignancy.

### 3.3. PTHrP and Medulloblastoma Cells

Medulloblastoma, an embryonal neuroepithelial tumor of the cerebellum, is a very common malignant tumor of the central nervous system in children, also displaying low survival rates. In 2007, Gessi *et al.* [37] produced evidence of high constitutive secretion of PTHrP by DAOY medulloblastoma cells. On the other hand, cell treatment with PTHrP antisense oligonucleotides resulted in a drastic decrease of cell proliferation and in the activation of the apoptotic program with an increase of *Fas* and *caspase 3* transcription rates, Bax/Bcl-2 ratio and intracellular Ca^++^ levels. This suggested that PTHrP could promote tumor growth, protecting cells from apoptosis, and, in addition, that it could be taken into consideration as a possible target for antisense strategy aimed to the control of medulloblastoma development.

### 3.4. PTHrP and Adrenocortical Tumor Cells

Adrenocortical cancer is a rare tumor with poor prognosis. Recently, Rizk-Rabin and collaborators [38] examined the relationship between *PTHrP* expression levels and clinico-pathological parameters of adrenocortical tumor and adrenal adenoma cells in a number of surgical specimens, the high activity of the gene resulting to be a selective hallmark of malignancy. Dealing with intracellular signalization in H295R cancer cells, they additionally reported that the protein functioned as an activator of cell cycle, releasing cells from G_1_/S checkpoint and consequently increasing the number of cells in the S and G_2_/M phases, and that PTHrP produced its action via two signaling pathways, intracellular cAMP/protein kinase A and Ca^++^/phospholipase C. Moreover, a positive correlation between protein production and steroid secretion was also proven. Incubation with anti-PTHrP antibody restrained cell survival and proliferation inducing the onset of apoptosis at day 5 from the beginning of treatment, as revealed by annexin binding assay. These cumulative data prompted further investigation on the intracellular biochemical cascade activated by PTHrP in this specific neoplastic cytotype.

### 3.5. PTHrP and Oral Squamous Cancer Cells

Yamada and coworkers [39] reported that in oral squamous cell carcinoma, a prevalent malignant tumor of the head and the neck, activation of EGFR induces via ERK and p30 MAPK *PTHrP* expression, which, in turn, activates ERK and Akt signalling via PTH1R in a paracrine/autocrine manner, leading to a promotion of tumor malignancy. In light of the observation that the functional inhibition of EGFR by AG1478, PTHrP silencing by RNA interference (RNAi), and combined application of these reagents exerted a prominent anti-tumoral effect, these findings raise the possibility to utilize both PTHrP and EGFR as therapeutic targets in oral squamous cell carcinoma.

### 3.6. PTHrP and Renal, Colon and Prostate Cancer Cells

Apart from the examples described in the previous paragraphs, most investigation on the proliferation-promoting and anti-apoptotic role played by PTHrP has been performed on cell model systems from these three tumor histotypes.

Massfelder and collaborators [40,41] found that PTHrP acts as an essential *in vitro* survival and growth factor for clear cell renal carcinoma lines which underwent apoptosis in the presence of PTHrP-neutralizing antibodies and following inhibition of PTH/PTHrP receptor. Interestingly, antibody treatment caused significant tumor regression in nude mice. Moreover, *PTHrP* expression and protein synthesis were found to be suppressed by the von Hippel-Lindau tumor suppressor protein, which is a component of an E3 ubiquitin ligase complex that targets the α subunits of HIF-1 and HIF-2 (hypoxia-induced factors) transcription factors for destruction in the presence of oxygen. When the latter one is functionally-inactivated, as occurs in 40–80% of conventional renal cell cancers, HIF-regulated genes, which code for several metabolic, angiogenic and growth factors, are over-expressed, thereby affecting a number of tumorigenesis-related biological activities. As the authors state, clear cell renal cancer is refractory to currently therapies and therefore, in light of the necessity for new agents for therapeutic intervention, the PTHrP/receptor system may be regarded as a promising target, consequently prompting further investigation on the molecular basis of PTHrP control of cell survival. Interestingly, Danilin and coworkers [42] recently documented the involvement of the mRNA-binding protein HuR in mRNA stabilization and increased expression of *PTHrP* in clear renal cell carcinomas. Additional results by Agouni and collaborators [43] showed that the phosphoinositide 3-kinase (PI3K)-Akt signaling is a key pathway for PTHrP-induced anti-apoptotic effect, in which integrin-linked kinase (ILK) is responsible for Akt phosphorylation which, in turn, activates the NFκB transcription factor. Thus, in renal cell carcinomas targeting of the PI3K/ILK/Akt/ NFκB axis may prove therapeutically beneficial.

Similar results were reported by Falzon and collaborators [44] in a study on human prostate cancer cell lines. It is known that prostate carcinogenesis is characterized by the increase of PTHrP secretion [45] which was proven to up-regulate integrin α6β4, capable to activate the PI3K pathway when signaling synergistically with growth factor receptors such as ErbB2, ErbB3, and c-Met. Thus, the PI3K-Akt axis was found to be involved also in the anti-apoptotic effect of PTHrP on this tumor cytotype. Moreover, PTHrP-mediated increase of the ratio of antiapoptotic (Bcl-2 and Bcl-xL) to proapoptotic (Bax) proteins as well as of the levels of Bad protein was also observed, and the development of strategies targeting PTHrP production in prostate cancer cells suggested therapeutic benefits. An alternative mechanism of proliferation-promoting action by PTHrP (1–34) has been proposed by DaSilva and coworkers [46] who demonstrated that under low androgen concentration LNCaP prostate cancer cells exposed to PTHrP up-regulate the androgen receptor (AR) via epidermal growth factor receptor and Src kinase activation-controlled restraining of proteosomal AR degradation. The proliferative advantage of PTHrP-treated cells might, therefore, result from over-expression of androgen-regulated genes. This study suggested potential new targets for therapeutic intervention. Noteworthy, Asadi and collaborators [47] have recently reported that adenovirus E1A oncogene represses *PTHrP* expression by PC3 prostate cancer cells, thereby decreasing cell survival due to the sensitization to apoptosis. This result could represent a promising starting point for the validation of E1A and/or other modulators of *PTHrP* expression, as activators of drugs considered *per se* ineffective in addressing prostate cancer cells to death.

Concerning colon cancer, PTHrP was proven to increase survival, migration and invasion of LoVo cells. Akt activation by phosphorylation and the consequent decrease in active glycogen synthase kinase-3 levels were found involved in such effect, which was most likely initiated by up-regulation of *integrin α6β4.* Moreover, PTHrP significantly increased LoVo cell anchorage-independent growth in soft agar via its intracrine pathway and nuclear action, and also induced xenograft growth in nude mice, thereby confirming its transformation potential also in *in vivo* models. More recently, up-regulation of *integrin α6β4* by PTHrP was proven to promote the migratory and invasive effect through activation of the Rho GTPase Rac1, known to play a role in cell motility [48], via the switching-on of the phosphatidylinositol 3-kinase/Akt pathway with the consequent activation of the Rac-specific guanine nucleotide exchange factor Tiam1 [49-52]. By consequence, targeting *PTHrP* expression and/or intracrine signaling might be a useful tool for the control of colon tumor cell growth, migration, and invasion.

Interestingly, the PTHrP-mediated over-expression of *α5* and *β1 integrin subunits* was reported in another colon adenocarcinoma cell line, HT-29, and found responsible for the increase of adhesion to extracellular matrix proteins (type I collagen, fibronectin), that was reverted by *PTHrP* silencing. Evidence was also brought that PTHrP regulates the expression level of *α5 integrin subunit* at a transcriptional level, although the direct or indirect interaction between the protein and the sequence of integrin promoter is still to be determined [53].

## 4. Conclusions: PTHrP, Immunotherapy and More

It is known that PTHrP can be regarded as a valuable prognostic factor of hypercalcemia-and tumor-affected patients. In light of such consideration, attempts were made to target it in anti-cancer treatments, especially when bone metastases developed. Harada and collaborators, who had identified protein fragments suitable for immunotherapy of HLA-A24^+^ and HLA-A2^+^ prostate cancer patients [54,55], in 2005 reported that C-terminal domains of PTHrP could be successfully used also to induce *in vitro* peptide-specific and cancer-reactive cytotoxic T lymphocytes from blood cells of gastric, colon and cervical cancer-affected patients, which could allow the design of specific vaccines [56]. More recently, the combination of anti-PTHrP treatment and zoledronic acid, a third generation bisphosphonate, was shown to better control the progression of bone metastases in natural killer cell-depleted immunosuppressed mice inoculated with SBC-5 human small cell lung cancer cells than either agent alone, thereby suggesting the usefulness of such dual-target therapy in treating lung tumor-affected patients [57].

In conclusion, the collective data briefly discussed in this paper strongly suggest that immunological or biomolecular therapeutic modulation of PTHrP may be a promising approach for establishment of anti-cancer strategies, and that elucidation of the mechanisms underlying its effects in the different cancer histotypes may identify useful targets for the design of novel anti-neoplastic drugs.
